# The Level of Cholesterol in COPD Patients with Severe and Very Severe Stage of the Disease

**DOI:** 10.3889/oamjms.2016.063

**Published:** 2016-05-24

**Authors:** Beti Zafirova-Ivanovska, Jagoda Stojkovikj, Dejan Dokikj, Sasha Anastasova, Angela Debresliovska, Sead Zejnel, Dragana Stojkovikj

**Affiliations:** 1 *Institute for Epidemiology and Biostatistics and Medical Informatics, Faculty of Medicine, Ss Cyril and Methodius University of Skopje, Skopje, Republic of Macedonia*; 2 *University Clinic of Pulmonology and Allergology, Faculty of Medicine, Ss Cyril and Methodius University of Skopje, Skopje, Republic of Macedonia*; 3 *University Clinic of Cardiology, Faculty of Medicine, Ss Cyril and Methodius University of Skopje, Skopje, Republic of Macedonia*; 4 *School of Doctoral Studies at Faculty of Medicine, Ss Cyril and Methodius University of Skopje, Skopje, Republic of Macedonia*

**Keywords:** severe and very severe COPD, comorbidity, the level of cholesterol, the level of LDL, the level of HDL

## Abstract

**BACKGROUND:**

High blood cholesterol is part of metabolic syndrome and can be caused by medical conditions or bad dietary habits.

**AIM:**

The aim of the study was to investigate the prevalence of hypercholesterolemia in privies diagnosed patients with the severe and very severe stage of COPD, which were stable.

**MATERIAL AND METHODS:**

We investigated 100 subjects, all of them smokers, with smoking status >10 years, stratified into two groups: with severe and very severe stage of the disease. It was clinical, randomized, cross-sectional study. Besides demographic parameters and functional parameters, body mass index, cholesterol, LDL, and HDL were investigated.

**RESULTS:**

In the group of patients with very severe COPD were recorded significantly higher average values of cholesterol (6.16 ± 1.5 vs. 5.61 ± 1.1, p = 0.039). As independent significant factors influencing cholesterol in the group with a very severe COPD were confirmed the age of the patients (p = 0.005), LDL (p = 0.004) and HDL (p = 0.002). In the group with severe COPD, only LDL was confirmed as an independent significant factor that has an impact on cholesterol (p < 0.0001).

**CONCLUSION:**

The results of our survey demonstrated a high level of blood cholesterol and LDL, and low level of blood HDL in both investigated group’s patients with COPD.

## Introduction

Chronic obstructive pulmonary disease (COPD) is characterized by minimally reversible or irreversible airflow limitation [1]. COPD is increasingly considered a multisystem disease characterized by both pulmonary and systemic inflammation. Pulmonary inflammation is a result of the abnormal inflammatory response of the lungs to noxious gasses and particles [1, 2]. The main epidemiological risk factor is smoking [3, 4]. Metabolic syndrome is defined as a complex of interrelated cardiovascular risk. High blood cholesterol and high triglycerides are part of metabolic syndrome and can be caused by medical conditions or bad dietary habits [5, 6].

Adipose tissue is now recognized not only as the main site of storage of excess energy derived from food intake but also as an endocrine organ. The expansion of adipose tissue produces a number of bioactive substances, known as adipocytokines or adipokines, which trigger chronic low-grade inflammation and interact with a range of processes in many different organs. Several inflammatory markers, such as TNF-α, PCR, IL-6, IL-8, Fas, Fas-L, Lipopolysaccharide Binding Protein have received great attention for their role in increased metabolism, weight loss, and asthenia although there is still no direct evidence for a cause-and-effect relationship between them. Systemic inflammation has become the primary focus to link COPD and cachexia and to explain the development of COPD as a syndrome in susceptible subjects [2, 7].

Current therapies for COPD have been shown to reduce symptoms and exacerbations and to improve the quality of life that is a therapeutic priority in these patients. However, these drugs have little effect on the natural history of the disease (progressive decline in lung function and exercise tolerance) and do not improve mortality [6]. Some authors recommended anti-inflammatory effects of statins on both pulmonary and systemic inflammation through inhibition of guanosine triphosphates and nuclear factor-κB mediated activation of inflammatory and matrix remodeling pathways could have substantial benefits in patients with COPD due to the following: 1) Inhibition of cytokine production (tumor necrosis factor-α, interleukin (IL)-6 and IL-8) and neutrophil infiltration into the lung; 2) inhibition of the fibrotic activity in the lung leading to small airways fibrosis and irreversible airflow limitation; 3) antioxidant and anti-inflammatory (IL-6 mediated) effects on skeletal muscle; 4) reduced inflammatory response to pulmonary infection; and 5) inhibition of the development (or reversal) of epithelial-mesenchymal transition, a precursor event to lung cancer. Common co-morbidities in COPD are cardiovascular diseases and they are associated with metabolic syndrome and they increased mortality risk in these patients [8-10].

A quarter of the world’s adults has metabolic syndrome. People with metabolic syndrome are twice as likely to die from, and three times as likely to have a heart attack or stroke compared with people without this syndrome [10].

According to the new International Diabetic Federation definition, for a person to be defined as having the metabolic syndrome must have: central obesity plus any two of the following four factors: raised triglyceride level, specific treatment for this lipid abnormality, reduced HDL cholesterol or specific treatment for this lipid abnormality, raised blood pressure, or treatment of previously diagnosed hypertension, raised fasting plasma glucose, or previously diagnosed type 2 diabetes, OGTT is strongly recommended but is not necessary to define presence of the syndrome [10-12]. In COPD patients, metabolic syndrome did not additionally impact patients’ functional outcomes but did impact the prevalence of co-morbidities first of all cardiovascular [13-15]. According to dates from some studies high level of cholesterol as a part of a metabolic syndrome in COPD patients is associated with more serious and more frequent exacerbations [16, 17].

The aim of the study was to investigate the prevalence of hypercholesterinemia [the level of cholesterol, HDL (high-density lipoproteins) and LDH (low-density lipoproteins)], in previously diagnosed COPD patients with severe and very severe stage of the disease.

## Material and Methods

We investigated 100 subjects with COPD, all of them smokers, with smoking status, >10 years. The duration of COPD in these patients was more than 9 years. They were stratified into two groups according to Global Initiative for Chronic Obstructive Lung Disease (GOLD). Sixty-four with severe stage of the disease: 50% > FEV1 ≥ 30%, FEV1/FVC < 0.70 and 36 subjects with very severe stage of the disease: FEV1 < 30%, FEV1/FVC < 0.70. It was clinical, randomized, cross-sectional study. Besides demographic parameters (age, gender), functional parameters, Body mass index (BMI) and the level of cholesterol, LDL and HDL were measured in blood.

### Statistical analysis

Statistical analysis of the database was made in the program SPSS for Windows 17.0. Testing of the distribution of the data was done with Kolmogorov - Smirnov and Shapiro-Wilk’s test. Categorical variables were presented with absolute and relative numbers; numeric variables were shown MPC descriptive statistics (mean, median, rank values). To test the significance of differences between the two COPD groups were used parametric and nonparametric methods for independent samples (Chi-square test, Student’s test, Mann-Whitney U test). To determine the correlation between cholesterol and certain parameters was used Pearson’s coefficient of linear correlation. Multiple regression analysis was used to determine significant independent factors associated with cholesterol. Statistically significant values were taken at p <0.05.

## Results

### Severe vs. very severe COPD

The results of our survey show that in male patients significantly more likely than in female patients were registered very severe COPD (45.45% vs. 17.65% p = 0.006) (Table 1).

**Table 1:** The prevalence of gender in bout groups of COPD patients

Gender	COPD (severe) N = 64	COPD (very severe) N = 36	p-value
Female n= 34	28 (82.35%)	6 (17.65%)	p = 0.006
Male n= 66	36 (54.55%)	30 (45.45%)	

p (Chi-square test) p < 0.01.

Patients with severe and very severe COPD were insignificant different average age and BMI (p = 0.75 and p = 0.14 consequently) (Table 2).

**Table 2:** The age, body mass index and functional parameters in bout groups of patients

Variable	Group	Mean ± SD	Min - Max	p-value
Age	Severe	62.53 ± 11.3	37 – 88	
	Very severe	61.83 ± 9.1	45 – 80	*p = 0.75
BMI (kg/m^2^)	Severe	24.22 ± 5.2	16 – 36	
	Very severe	25.9 ± 5.9	19.3 – 35	*p = 0.14
FVC (L)	Severe	1.78 ± 0.5	0.9 - 3.28	*p < 0.01
	Very severe	1.23 ± 0.4	0.56 -1.89	
FVC (%)	Severe	56.25 ± 11.2	40 – 88	*p < 0.01
	Very severe	37.67 ± 12.9	20 - 67	
FEV1 (L)	Severe	1.02 ± 0.2	0.64 -1.58	*p < 0.01
	Very severe	0.67 ± 0.2	0.25 - 0.53	
FEV1 (%)	Severe	40.6 ± 6.8	31-50	*p < 0.01
	Very severe	23.3±5.1	11-29	
FEV1/FVC (%)	Severe	60.84 ± 6.8	48 – 70	*p < 0.01
	Very severe	52.61 ± 8.6	36 – 66	

p (Student’s t- test).

In the group of patients with very severe COPD were recorded significantly higher average values of cholesterol (6.16 ± 1.5 vs. 5.61 ± 1.1 p = 0.039). The values of LDL and HDL were insignificant different in the group with severe and very severe COPD (p = 0.66 and p = 0.11 respectively) (Table 3).

**Table 3:** The level of cholesterol, LDL, and HDL in patients with severe vs. very severe COPD

Variable	Group	Mean ± SD	Min - Max	Median (IQR)	p-value
Cholesterol (mmol/l)	Severe	5.61 ± 1.1	3.7 – 8.7	5.60 (4.6 – 6.6)	*p =0.039
	Very sever	6.16 ± 1.5	4.3 – 9.6	5.85 (4.9 – 6.7)	
LDL (mmol/l)	Severe	3.10 ± 1.1	1.5 – 5.2	2.75 (2.1 – 3.9)	**p =0.66
	Very severe	3.46 ± 1.7	1.6 – 6.7	3.15 (1.9 – 4.5)	
HDL (mmol/l)	Severe	1.31 ± 0.6	0.4 – 2.2	1.45 (0.75 – 1.8)	**p =0.11
	Very severe	1.08 ± 0.6	0.4 – 2.2	0.85 (0.6 – 1.7)	

* p (Student-s t-test);

** p(Mann-Whitney U test).

### Cholesterol

Male with very severe COPD had significantly higher average cholesterol levels than female (5.85 ± 1.2 vs. 5.29 ± 0.9 p = 0.043). In the group with severe COPD, male patients had cholesterol values slightly higher than females (p = 0.13) (Table 4).

**Table 4:** The average value of cholesterol in female vs male in both groups

COPD group	Gender	Descriptive statistics-cholesterol			p-value

		mean ± SD	min - max	median (IQR)	
Severe	Female n=28	5.29 ± 0.9	3.7 – 6.8	5.35 (4.6 – 5.8)	*p = 0.043
	Male n=36	5.85 ± 1.2	4.3 – 8.7	5.65 (4.9 – 6.6)	
Very Severe	Female n=6	5.27 ± 0.8	4.6 – 6.3	4.95 (4.6 – 6.3)	**p = 0.13
	Male n=30	6.34 ± 1.6	4.3 – 9.6	5.90 (5.1 – 7.0)	

* p (Student-s t-test);

** p(Mann-Whitney U test).

In the group with severe COPD, cholesterol significantly positively correlated with LDL (r = 0.895, p < 0.001), and significantly negatively correlates with HDL (r = - 0.667, p <0.001).

In the group with very severe COPD, cholesterol significantly positively correlated with LDL (r = 0.895, p <0.001), while significantly negatively correlated with the age of patients (r = - 0.458, p = 0.005) and the values of FVC% (r = - 0.37, p = 0.025) and HDL (r = - 0.793, p <0.001) (Table 5).

**Table 5:** The correlation of cholesterol with age, functional parameters, BMI, cholesterol, LDL, HDL

Correlation cholesterol/	r - Pearson	

	Severe	Very severe
Age (years)	r = - 0.07, p=0.57	r = - 0.458, p=0.005
FVC (L)	r = 0.048, p=0.7	r = - 0.115, p=0.5
FVC (%)	r = 0.020, p=0.87	r = - 0.370, p=0.025
FEV1 (L)	r = 0.037, p=0.77	r = - 0.115, p=0.5
FEV1 (%)	r = - 0.057, p=0.66	r = - 0.315, p=0.06
FEV1/FVC	r = 0.070, p=0.57	r = 0.114, p=0.4
BMI (mmol/l)	r = 0.038, p=0.77	r = 0.230, p=0.18
LDL (mmol/l)	r = 0.895, p<0.001	r = 0.896, p<0.001
HDL (mmol/l)	r = - 0.667, p<0.001	r = - 0.793, p<0.001

Results from Multiple regression analysis which investigate the impact of LDL and HDL cholesterol in the group with severe COPD, (which bivariate analysis proved to be significantly associated with cholesterol), only LDL confirmed as an independent significant factor that has an impact on cholesterol (p < 0.0001). With the increase in LDL by 1 mmol/l, average values of the cholesterol increase of 0.927 (B = 0.927) (Table 6).

**Table 6:** Multiple Regression analysis of the impact of LDL and HDL cholesterol in the group with severe COPD Coefficients

Coefficients							

Model	Unstandardized Coefficients		Standardized Coefficients	t	Sig.	95% Confidence Interval for B	
	
	B	Std. Error	Beta			Lower Bound	Upper Bound
1 (Constant)	2.565	0.463		5.540	0.000	1.639	3.491
LDL	0.927	0.087	0.958	10.614	0.000	0.752	1.101
HDL	0.147	0.166	0.080	0.882	0.381	-0.186	0.479

a) Dependent Variable: cholesterol.

**Figure 1:**
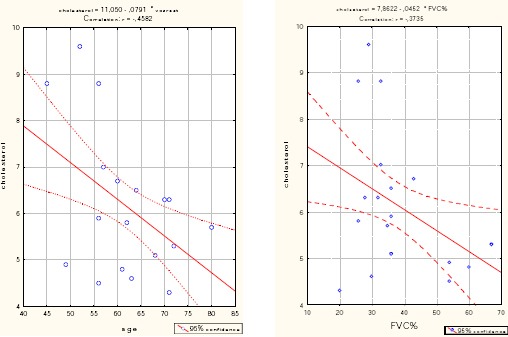
*Correlation - cholesterol vs. age (left) and FVC% (right) in the group with a very severe COPD*.

As independent significant factors influencing cholesterol in the group with a very severe COPD confirmed the age of the patients (p = 0.005), LDL (p = 0.004) and HDL (p = 0.002). With increasing age of one year, the average cholesterol was reduced by 0.044 (B = - 0.044).

**Figure 2:**
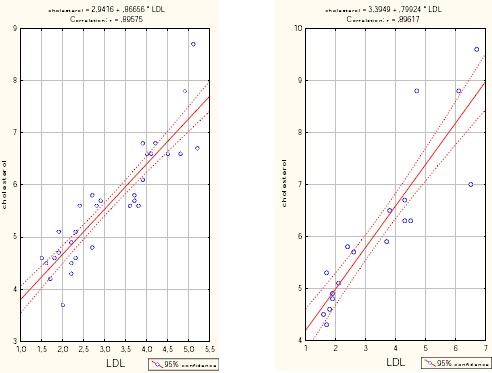
*Correlation cholesterol/LDL cholesterol correlated/LDL group severe COPD (left) and group very severe COPD (right)*.

With increasing values of LDL by 1 mmol/l, average values of the cholesterol increase of 0.399 (B = 0.399). With increasing values of HDL to 1 mmol/l, average values of cholesterol is reduced by 1.139 (B = - 1.139) (Table 7).

**Table 7:** Multiple regression analysis of the impact of age, FVC, LDL, HDL and blood glucose, cholesterol in the group with a very severe COPD Coefficients

Coefficients							

Model	Unstandardized Coefficients		Standardized Coefficients	t	Sig.	95% Confidence Interval for B	
	
	B	Std. Error	Beta			Lower Bound	Upper Bound
1 (Constant)	8.765	1.537		5.702	0.000	5.634	11.896
Age	-0.044	0.015	-0.258	-2.979	0.005	-0.075	-0.014
LDL	0.399	0.127	0.447	3.150	0.004	0.141	0.657
HDL	-1.139	0.342	-0.430	-3.332	0.002	-1.836	-0.443

a Dependent Variable: cholesterol

**Figure 3:**
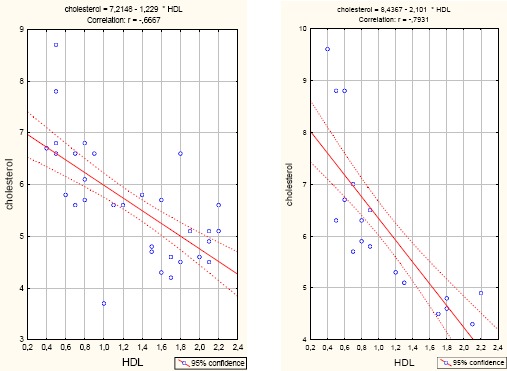
*Correlation cholesterol/HDL cholesterol correlated/HDL group severe COPD (left) and group very severe COPD (right)*.

## Discussion

Hypercholesterolemia in COPD is considered as comorbidity, one of the metabolic syndrome. According to some dates severe COPD is associated with increased levels of HDL-C, which is partially attributable to oral steroid use. HDL-C in this population is not associated with reduced risk of angiographically proven coronary artery disease [18].

In the Joseph Davaney study from 2014, metabolic syndrome was present in 57% of the COPD patients and 40% of the healthy subjects. After stratification for BMI, presence of metabolic syndrome in patients with a BMI ≥ 25 kg/m^2^ was higher than in healthy peers. Patients with metabolic syndrome and a BMI <25 kg/m^2^ had higher BMI, fat-free mass index, and bone mineral density, and a lower 6 MWD than the BMI-matched patients without metabolic syndrome. In COPD patients with metabolic syndrome self-reported comorbidities and medication use were higher than in those without [19]

In our survey in both groups of patients with the severe and very severe disease, BMI did not correlate significantly with cholesterol.

Metabolic syndrome did not additionally impact patients’ functional outcomes but did impact the prevalence of comorbidities. Metabolic syndrome is a common metabolic disorder defined as a complex of interrelated cardiovascular risk factors. Metabolic syndrome is age dependent and has been related to several other health conditions and an increased mortality risk [19].

In our survey in the group with very severe COPD, cholesterol significantly negatively correlated with the age of patients (r = - 0.458, p = 0.005). Men with very severe COPD had significantly higher average cholesterol levels than women (5.85 ± 1.2 vs. 5.29 ± 0.9, p = 0.043). In the group with severe COPD, male patients had cholesterol values slightly higher than females (p = 0.13).

The prevalence of metabolic syndrome in COPD patients compared to healthy subjects has been studied scarcely. Marquis and colleagues reported an increased prevalence in 38 COPD patients compared to 34 healthy subjects (47% vs. 21%, respectively). A comparable prevalence of metabolic syndrome in COPD was reported by Watz and colleagues, but no healthy control group was included in this study [10, 11, 17].

COPD patients with metabolic syndrome are physically less active and have increased levels of systemic inflammation compared to COPD patients without metabolic syndrome. Low-grade systemic inflammation has been present in participants with moderate to severe airflow obstruction and was associated with increased risk of cardiac injury. This may in part explain the high rates of cardiovascular complications in COPD [19, 20].

A number of studies have shown an association between COPD and selected CVD end points including total cardiac mortality, mortality from acute myocardial infarction (AMI), mortality after coronary artery bypass graft, and pulmonary embolism. Low FEV1 is associated with all-cause mortality, CVD mortality, nonfatal and fatal myocardial infarction (MI), nonfatal and fatal stroke, and atrial fibrillation [21].

In our survey in the both groups patients with COPD, cholesterol significantly positively correlated with LDL (r = 0.895, p <0.001), (r = 0.896, p <0.001) and significantly negatively correlates with HDL (r = - 0.667, p <0.001); r = - 0.793, p<0.001).

Osteoporosis is common in advanced COPD, potentially impairing quality of life. Increased high-density lipoprotein cholesterol [HDL-C (high-density lipoprotein)] has been observed in COPD and linked with osteoporosis in the general population. This association has not been previously examined in COPD [22].

Subjects with COPD who perform some level of regular physical activity have a lower risk of both COPD admissions and mortality. The recommendation that COPD patients are encouraged to maintain or increase their levels of regular physical activity should be considered in future COPD guidelines since it is likely to result in a relevant public health benefit [23].

Some dates from recent studies suggest that exercise can elevate HDL-C. Patients with chronic obstructive pulmonary disease (COPD), who are usually considered as leading sedentary lifestyles, have an increased work of breathing and their respiratory muscles may be considered to be under a chronic exercise load. Severe COPD is associated with increased levels of HDL-C, which is partially attributable to oral steroid use. HDL-C in this population is not associated with reduced risk of angiographically proven coronary artery disease [24, 25].

In conclusion, COPD is considered as a systemic disease according to much concomitant comorbidity in patients. These comorbidities significantly impact on patient outcomes. Evidence for this approach has been provided by strong associations with increased rates especially with cardiovascular diseases, metabolic syndrome, anemia, musculoskeletal disease and pulmonary malignancies. A number of studies have shown a high connectivity between COPD and cardiovascular morbidity and mortality and pulmonary embolism. Hypercholesterinemia probably is hugely responsible for those events. The results of our survey show a high level of blood cholesterol and LDL, and low level of blood HDL in both investigated group’s patients with COPD. Patients with very severe COPD have significantly higher average values of cholesterol (6.16 ± 1.5 vs. 5.61 ± 1.1, p = 0.039). The values of LDL and HDL were insignificant different in the group with severe and very severe COPD (p = 0.66 and p = 0.11 respectively).
